# Impact of Chemoradiotherapy on Quality of Life in Cervical Cancer Patients: A Prospective Cohort Study

**DOI:** 10.3390/jcm14176023

**Published:** 2025-08-26

**Authors:** Maria-Alexandra Barbu, Miruna Ghigeanu, Sarah Bahaa-Eddin, Alexandru Michire, Alexandra Hanu, Gentiana Eremia, Ianina Draganescu, Alina Birca, Mihai-Teodor Georgescu

**Affiliations:** 1 Department 8—Radiology, Oncology, Hematology, Faculty of Medicine, “Carol Davila” University of Medicine and Pharmacy, Bulevardul Eroii Sanitari 8, 050474 Bucharest, Romania; maria.barbu@umfcd.ro; 2 MedEuropa Center of Oncology and Radiation Therapy, Dobroesti 20a, 022343 Bucharest, Romania; 3 Oncology Department, Colțea Clinical Hospital, Ion C. Brătianu 1, 030167 Bucharest, Romania; 4 Radiotherapy Department, Sanador Oncology Center, Strada Sevastopol, Nr 4, Sector 1, 010993 Bucharest, Romania; 5 Prof. Dr. Matei Balș Institute of Infectious Diseases, 021105 Bucharest, Romania; 6 Prof. Dr. Al. Trestioreanu Oncology Institute, Radiotherapy 2 Department, 022328 Bucharest, Romaniamihai.georgescu@umfcd.ro (M.-T.G.); 7 Prof. Dr. Al. Trestioreanu Oncology Discipline, Department 8, Faculty of Medicine, “Carol Davila” University of Medicine and Pharmacy, 022328 Bucharest, Romania

**Keywords:** cervical cancer, chemoradiotherapy, quality of life (QoL), Functional Assessment of Cancer Therapy-General (FACT-G), adverse events

## Abstract

**Background**: Cervical cancer is considered to be a global health challenge, particularly in low- and middle-income countries. Romania reports one of the highest burdens in Europe due to limited access to screening and HPV vaccination. Chemoradiotherapy is standard for locally advanced disease, but the impact on quality of life (QoL) for a low- and middle-income population has not yet been explored. This study aims to evaluate the effect of chemoradiotherapy on the QoL of cervical cancer survivors in the Romanian population. **Methods**: This prospective observational study included 111 patients with stage I–IV cervical cancer undergoing chemoradiotherapy. QoL was assessed using the Functional Assessment of Cancer Therapy-General (FACT-G) questionnaire before, during, and after treatment. Demographic and clinical data were collected. The statistical analyses included t-tests, ANOVA, and linear mixed-effects models to evaluate changes over time and the influence of sociodemographic and treatment-related factors. **Results**: FACT-G scores significantly increased after treatment, with improvements in physical and functional well-being. Better before-treatment QoL was associated with urban residence, early-stage disease, marital status, and higher education. Among treatment toxicities, only nausea had a statistically significant negative effect on QoL during treatment, while other toxicities showed no significant impact. **Conclusions**: Chemoradiotherapy in cervical cancer patients was not associated with a substantial deterioration in quality of life during treatment and was followed by significant improvement after therapy completion. These findings highlight a favorable short-term QoL trajectory and emphasize the need for longitudinal studies to assess whether such benefits are maintained over the long term.

## 1. Introduction

Cervical cancer (CC) remains a significant global health challenge, ranking as the fourth most common cancer among women. According to GLOBOCAN 2024 data [1], approximately 660,000 new cases and 350,000 deaths were recorded worldwide, with 90% of cases occurring in low- and middle-income countries due to limited healthcare access, early screening, and HPV vaccination coverage.

In Europe, cervical cancer poses a substantial public health issue, with over 66,000 new cases and 30,000 deaths annually. Disparities in healthcare infrastructure, public health funding, and cultural factors influence the effectiveness of screening and vaccination programs. Romania has one of the highest cervical cancer burdens in Europe, with 3368 new cases and 1793 deaths annually. Approximately 11,278 women are living with cervical cancer, highlighting the urgency of enhancing early detection, care access, and HPV vaccination efforts [2]. These statistics underscore the critical need for preventive measures, including widespread HPV vaccination and effective screening programs, to reduce the burden of cervical cancer in Romania and across Europe [3].

Due to advancements in treatment and screening, CC is often diagnosed earlier, resulting in improved survival rates, with a 5-year relative survival rate of 91.5% in the early stages [4]. Around 67.2% of women diagnosed with cervical cancer survive for five or more years after diagnosis. In Europe, the net survival rate is approximately 64%. It is important to note that early detection of cervical cancer significantly improves survival rates, as early-stage diagnoses offer a better prognosis [5].

HPV infection accounts for 90–100% of cervical cancer cases, particularly in women under 35 years [6]. Treatment strategies, including surgery, radiation, and chemotherapy, depend on the disease stage and may involve combinations of these modalities [7]. Radiation therapy, often paired with chemotherapy, is the standard for advanced cases [8], while drugs like Cisplatin and Paclitaxel demonstrate consistent efficacy [9].

Survivors often face physical and psychosocial challenges, with chemoradiotherapy causing side effects like bladder and bowel dysfunction, which can greatly influence health-related quality of life (HRQoL). Patient-centered care and QOL assessment tools, endorsed by the WHO, are essential for improving treatment outcomes [10,11].

## 2. Materials and Methods

This prospective observational study included women diagnosed with cervical cancer at our clinics during a one-year period from January 2023 to January 2024, all of whom received chemoradiation treatment.

**Figure 1:**
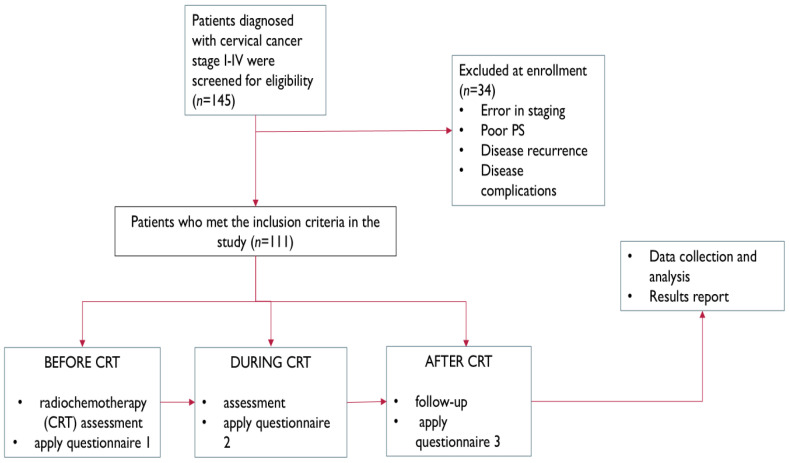
Study flowchart.

The data was collected in accordance with ethical guidelines to ensure patient confidentiality. Patients observed over a one-year period were included based on eligibility for chemoradiotherapy, defined as ECOG performance status 0–2 and FIGO stage I–IV cervical cancer. Exclusion criteria comprised recurrent disease and complications likely to interfere with treatment or quality of life assessment, including urinary tract obstruction, vesicovaginal or rectovaginal fistulas, severe pelvic pain requiring opioids, lower limb lymphedema, active infection, or significant vaginal bleeding. We have attached the corresponding flow chart, as seen as in Figure 1.

Data was collected using assessments conducted at baseline (pre-treatment), during treatment, and at a follow-up point after treatment completion to measure changes in QoL over time. Parameters assessed included demographic information (age, education level, and residence), histopathological subtype, disease stage at diagnosis, radiotherapy dose, chemotherapy type, number of brachytherapy insertions, and adverse events during treatment with corresponding grades.

Quality of life (QoL) was assessed using the Functional Assessment of Cancer Therapy-General (FACT-G) questionnaire, which evaluates multiple scales of well-being: Physical Well-Being (PWB), Social Well-Being (SWB), Emotional Well-Being (EWB), and Functional Well-Being (FWB). Scores on these scales provide insight into the broader impacts of treatment beyond clinical outcomes. Baseline QoL was compared to post-treatment scores to assess the impact of chemoradiation on patients’ quality of life [12].

The primary treatment for cervical cancer patients in this cohort was chemoradiation. Radiotherapy protocols varied according to cancer type and intent of treatment. The total radiotherapy dose varied according to FIGO stage. Patients with early-stage disease (stage I) typically received 45–50.4 Gy EBRT with no boost. In more advanced stages (II–IV), a tumor boost was added (either as simultaneous integrated boost or sequentially), with total EBRT doses reaching up to 59.4 Gy. Brachytherapy doses regimens varied from 3 to 5 fractions and ranged between 5.5 and 7 Gy per fraction. The cumulative EQD2 ranged from approximately 75 Gy in the early stages to around 85 Gy in stage III–IV patients.

Image-guided brachytherapy (CT-based) was administered in 3–5 sessions, with a mean dose of 6.5 Gy per fraction (range: 5.5–7.5 Gy). Concurrent chemotherapy was administered with weekly cisplatin at a dose of 40 mg/m^2^, or, in select cases, carboplatin dosed at AUC 2, according to patient tolerance and clinical indications.

Descriptive statistics, including means, standard deviations, and frequency distributions, were calculated for demographic and clinical characteristics using Microsoft Excel 2023 and RStudio 2023.12 “Ocean Storm” with ggplot2 and tidyverse packages [13,14,15,16]. Comparisons between pre- and post-treatment QoL scores were conducted using t-tests for continuous variables, one-way/two-way ANOVA for multiple comparisons with post hoc test Tukey HSD, and chi-square tests to assess associations between categorial variables. Statistical significance was set at *p* < 0.05.

To evaluate the impact of treatment-related toxicities on patient-reported quality of life, we conducted a series of linear mixed-effects models using the lmer function in the lme4 package in R [17]. The primary outcome was the total score on the FACT-G questionnaire, measured before treatment and at two treatment timepoints (during and after treatment). Each model included one binary toxicity variable (e.g., nausea, dysuria, diarrhea, hematological adverse events) as a fixed effect, along with timepoint and their interaction, and a random intercept for patient ID to account for within-subject correlation. The main effect of each toxicity represents the difference in FACT-G score at T1 between patients with and without that specific symptom. The interaction term (timepoint × toxicity) tested whether the change in FACT-G score from T1 to T2 differed by toxicity status. All models were fit using restricted maximum likelihood (REML). Effect estimates and 95% confidence intervals were extracted and visualized to compare the relative impacts of each toxicity on baseline quality of life and their trajectories over time.

## 3. Results

This study analyzed a total of 111 patients diagnosed with cervical cancer, ranging in age between 29 and 82 years old, with a mean of 58 years. Most patients were diagnosed with squamous cell carcinoma (*n* = 81), followed by adenocarcinoma (*n* = 27), and three patients were diagnosed with neuroendocrine carcinoma.

### 3.1. Patient Characteristics

As presented in Table 1, most patients were married at the time of treatment, lived in urban areas, and had middle levels of income. In total, 50% of patients had stage III cervical cancer, and 34% had stage II. The most used chemotherapy agent was cisplatin, administered to 92% of patients. There were five grade 3 toxicities and four grade 4 toxicities. The most common toxicities were nausea, diarrhea, and emesis. Eight patients had grade 1 ototoxicity and four had grade 1 renal toxicity.

A limitation of this study is that chemotherapy records were incomplete for nine patients (8.1%), preventing the accurate identification of the radio sensitizing chemotherapy drugs used. However, given that these nine patients represent a small proportion of the total cohort (8.1% of one hundred and eleven patients), this did not have a statistically significant impact on the overall quality of life (QoL) analysis. While these patients were included in the general QoL assessments, no other subgroup analysis was conducted for this group. As a result, this may introduce a minor bias when interpreting the treatment-related effects on QoL.

Regarding brachytherapy, not all patients received it at the time of evaluation or opted out of receiving it, hence the number mismatch.

#### Interpretation of FACT-G Score

**Figure 2:**
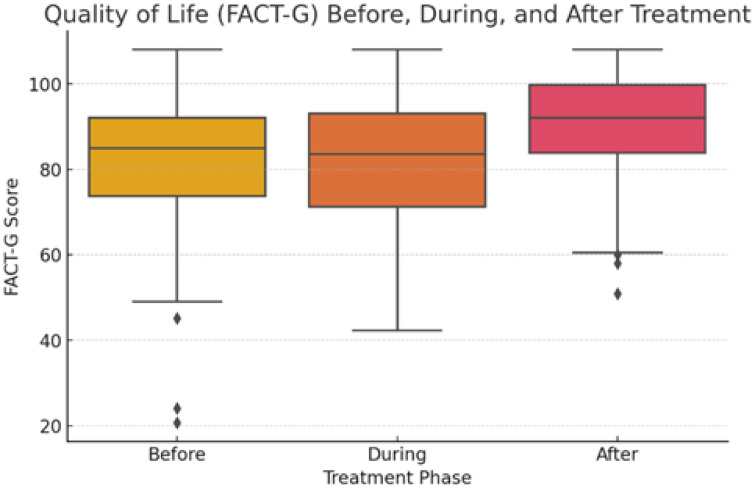
FACT-G subdomain scores before, during, and after treatment.

The median general quality of life score (FACT-G) before treatment was 85 (IQR 73–92), during treatment was 84 (71–93), and after treatment was 92 (84–100). The scores are summarized in Table 2, along with the subdomains. The FACT-G score increased significantly after treatment (F-statistic = 13.05, *p*-value < 0.001). This difference was significant between the score before and after treatment (Tukey’s HSD *p* < 0.001) and for during and after treatment (*p* < 0.001, Figure 2).

The effect size for overall FACT-G improvement was moderate, with Cohen’s d = 0.61 for before vs. after treatment and d = 0.63 for during vs. after treatment.

### 3.2. Observations from Each Subdomain

**Figure 3:**
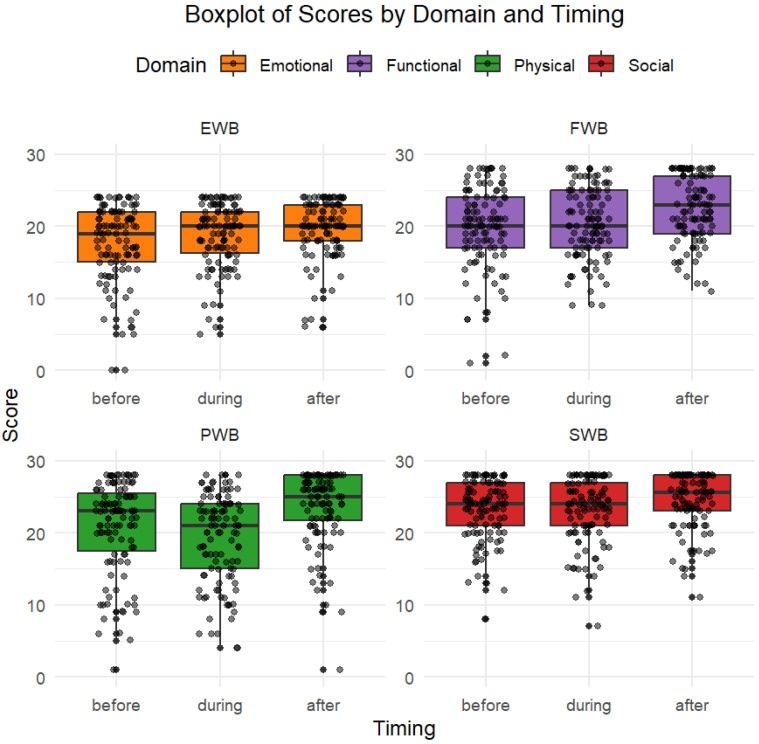
Quality of life for each subdomain. Overall, the score increases for each subdomain after treatment, with slightly worse scores during treatment.

Physical Well-Being (PWB) median score increased after treatment compared to before and during treatment (25 points vs. 23 and 21, *p* = 0.0017). The effect size for PWB was moderate to large (Cohen’s d = 0.48 before vs. after; d = 0.75 during vs. after). Social Well-being (SWB) was slightly better after treatment than before, although not statistically significant (*p* = 0.068), with a small effect size (Cohen’s d = 0.31). Emotional Well-Being (EWB) slightly decreased during treatment and returned to the initial value after treatment (19 points during and 20 points before and after treatment), with a small effect size (Cohen’s d = 0.20 during vs. after; d = 0.44 before vs. after). Functional Well-Being (FWB) improved significantly after treatment (23 points after vs. 20 during and before, *p* < 0.001), with a moderate effect size (Cohen’s d = 0.50 before vs. after; d = 0.45 during vs. after). Overall, the score increases for each subdomain after treatment, with slightly worse scores during treatment, as seen in Figure 3.

### 3.3. Influence of Sociodemographic Factors

Patients from urban areas had a baseline FACT-G score 8.3 points higher than patients in rural areas (*p* < 0.001, 95% CI [5.07–11.54]), while single status vs. married status showed a difference of 6.59 points in the baseline scores (*p* = 0.004, 95% CI [0.26–12.93]). Patients with stage I–II had a higher score than patients with stage III–IV, with a difference of 4.43 points (*p* = 0.046, 95% CI [1.38–7.5]). For EWB scores, only the area associated a difference in the baseline score (2.19, *p* = 0.045, 95% CI [0.05–4.35]). For PWB, education was associated with a better score in patients with middle education vs. elementary education (4 points difference, *p* = 0.0014, 95% CI [1.5–7.4]).

Sociodemographic factors significantly influenced baseline quality of life: patients living in urban areas, those with early-stage cancer, and married individuals reported higher overall FACT-G scores.

Physical Well-Being was positively associated with education, living environment, and stage. Functional Well-Being was strongly influenced by area of residence, and Emotional Well-Being was modestly affected by living environment. Social Well-Being showed no significant sociodemographic associations.

### 3.4. Effects of Chemotherapy and Toxicities on Quality of Life

Ninety-four patients had cisplatin, eight had carboplatin, and nine had unknown chemotherapy agents. For pre-treatment FACT-G score, ANOVA yielded an F-value = 0.705 (*p*-value = 0.496), post hoc analysis showed a 0.47 difference between cisplatin and carboplatin (*p* = 0.99), unknown—cisplatin of 6.58 (*p* = 0.47) and unknown—carboplatin 7.1 (*p* = 0.63). For treatment FACT-G, F-value = 0.94 (*p* = 0.39) with cisplatin–carboplatin having a difference of 2 points (*p* = 0.93), unknown—carboplatin 8.6 (0.45), and other—cisplatin 6.6 (*p* = 0.4). After treatment, F-value = 2.87 (*p* = 0.06) with 4.2 points difference for cisplatin–carboplatin, carboplatin—unknown 5.5 (*p* = 0.64), and cisplatin—unknown 9.8 (*p* = 0.06). Despite numeric differences between the scores, the results were not statistically significant, probably because the majority of the patients received cisplatin.

There was a main effect of time with better FACT-G scores from baseline to after treatment. There was a main effect of cancer at baseline and mid-treatment, patients with stage I–II having a better QOL than patients with stage III–IV (difference of 4.43 points, *p* = 0.0046). The interaction between stage and time was not statistically significant.

**Figure 4:**
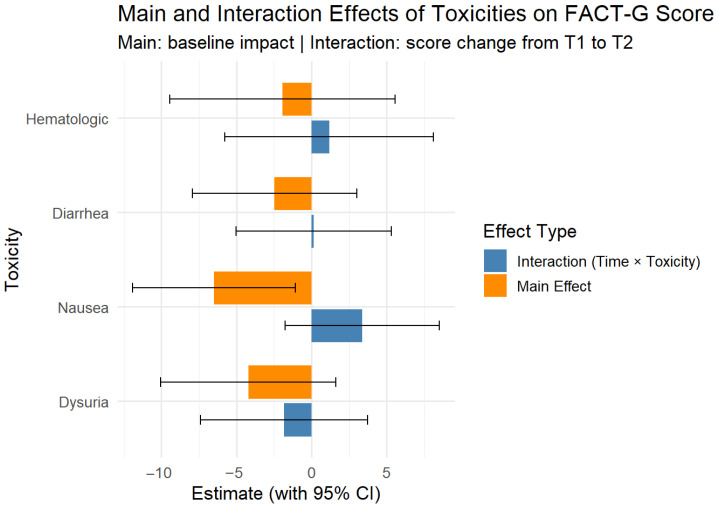
Mixed effects for FACT-G score at baseline (T1) to after treatment (T2) for main toxicities (binary). Bars not crossing 0 are statistically significant.

**Figure 5:**
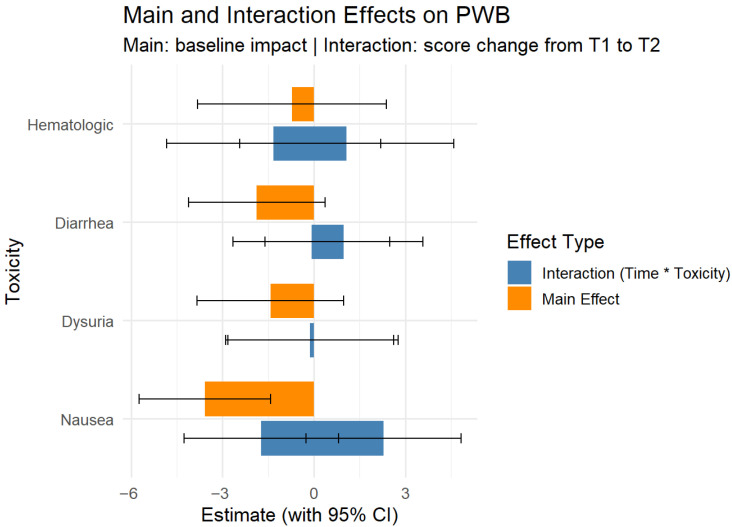
Mixed effects for PWB scores at baseline (T1) to after treatment (T2). Bars not crossing 0 are statistically significant.

Regarding the effects of toxicity on scores (toxicity levels can be seen in Table 3), only nausea was statistically significant; patients with nausea had lower FACT-G score during the treatment compared to those without nausea (β = −6.49, 95% CI [−11.91, −1.07], Figure 4). Patients with nausea also had lower PWB scores during treatment than patients without (β= −3.58, 95% CI [−5.74, −1.41]). The other toxicities showed associated lower scores, but they were not statistically significant on any of the subdomains (Figure 5).

Linear mixed-effects models revealed that, among treatment-related toxicities, only nausea had a statistically significant impact on quality of life, being associated with lower Physical Well-Being (β = −3.58, 95% CI [−5.74, −1.41]). Other toxicities, including hematologic effects and dysuria, did not significantly influence any subdomains of the FACT-G score. No significant interaction effects were found.

## 4. Discussion

Cervical cancer (CC) treatments, particularly chemoradiation, profoundly influence patients’ quality of life (QoL). This study aims to evaluate the effects of chemoradiation on the Physical, Social, Emotional, and Functional Well-Being of women with cervical cancer.

### 4.1. Impact of Cervical Cancer Chemoradiation on Quality of Life

In this prospective cohort, we report that chemoradiotherapy for cervical cancer was associated with no substantial deterioration in overall quality of life during treatment, followed by a significant improvement in quality of life after the completion of therapy. Median FACT-G scores decreased only slightly from baseline to mid-treatment, with the decline not reaching a clinically significant magnitude, and then improved significantly at post-treatment assessment. This pattern was consistent across most subdomains, with Physical and Functional Well-Being showing the most marked gains after treatment. Instead, patients maintained their baseline QoL and subsequently reported meaningful improvements following treatment. This trajectory contrasts with several prior reports describing more pronounced treatment-related declines in QoL.

### 4.2. Physical and Functional Impact of Chemoradiation

While chemoradiation is an effective treatment for cervical cancer, it often causes significant adverse effects that negatively affect QoL [18,19,20]. This study identifies several treatment-related toxicities, including dysuria, nausea, vomiting, fatigue, pain, gastrointestinal symptoms, and hematologic complications. These physical challenges often led to declines in functional well-being, impairing daily activities and social interactions. These findings indeed align with previous research [21], which highlights the negative physical effects of chemoradiation. As seen in the specialty literature [21,22,23], our study also emphasizes importance of minimizing these side effects to improve patient outcomes.

### 4.3. Influence of Demographics and Disease Characteristics

The study reveals that QoL outcomes varied according to demographic- and disease-related factors. Older age and advanced disease stages were linked to substantial declines in Physical and Functional Well-Being, while higher education levels were associated with better Social Well-Being. These results align with earlier findings demonstrating the impact of socioeconomic and demographic factors—such as cancer stage, treatment type, and social support—on treatment outcomes and recovery [4].

### 4.4. Treatment Modalities and QoL Outcomes

In our study, chemoradiotherapy was associated with only a slight, non-clinically meaningful decline in QoL during treatment, followed by a significant improvement after completion, suggesting that the overall trajectory of patient-reported outcomes was favorable compared to the more substantial long-term impairments described in some previous reports. Our study focuses on acute and medium-term QoL changes, contrasting with earlier research that noted persistent QoL declines up to ten years post-treatment [24]. Adverse events related to cisplatin-based chemoradiation, such as hematologic, gastrointestinal, and renal toxicities, have been well described over the past decades, with our study also further aligning with the literature.

Their impact on patient-reported quality of life, however, appears to vary according to the PROM used. Studies employing the EORTC QLQ-C30 and the disease-specific module QLQ-CX24 have typically reported significant impairments in physical and role functioning during treatment, with gradual recovery within 6–12 months [4]. In contrast, FACT-G–based studies, including ours, often show only modest declines during treatment, with significant improvements post-therapy [23].

These differences may reflect variations in sensitivity between instruments, timing of assessment, and supportive care practices, which should be considered when comparing QoL outcomes across cohorts.

Expanding future analyses to include long-term follow-ups [24,25,26] may provide deeper insights [23].

### 4.5. Importance of QoL Assessments in Routine Care

Integrating QoL assessments into routine care for cervical cancer patients undergoing chemoradiation is essential. Identifying patients at risk of significant QoL declines enables timely interventions, such as managing adverse events and educating patients about potential treatment challenges. In our study, we state that supportive care strategies, including counseling and rehabilitation, can help alleviate the physical, emotional, and social burdens associated with treatment.

### 4.6. Advancements in Radiotherapy

In line with these technological improvements, our cohort received contemporary chemoradiotherapy and image-guided brachytherapy. Although numeric differences in QoL scores were observed according to the chemotherapy agent received, these did not reach statistical significance, likely because the majority of patients were treated with cisplatin as the radiosensitizing drug.

The results show no substantial QoL deterioration during treatment and a significant improvement afterwards, highlighting the potential benefit of modern radiotherapy techniques in mitigating treatment-related burden.

Previous studies report, in concordance with ours, that radiotherapy remains a cornerstone for managing locally advanced cervical cancer, particularly when combined with brachytherapy [27]. Modern approaches, such as intensity-modulated radiation therapy (IMRT), have demonstrated reduced gastrointestinal and genitourinary toxicity compared to traditional methods, enhancing patients’ treatment experiences [28]. However, side effects, such as diarrhea, nausea, vomiting, dysuria, and peripheral neuropathy, remain common [29,30].

### 4.7. Socio-Demographic Factors and QoL

Consistent with our findings, sociodemographic characteristics such as urban residence, higher education, and marital status were associated with better baseline QoL and post-treatment outcomes, reinforcing the role of social and economic context in shaping patients’ quality of life trajectories during and after chemoradiotherapy. Conversely, lower income and unemployment are linked to poorer survival outcomes. Advanced disease stages at diagnosis further exacerbate QoL and survival disparities [31].

### 4.8. Combined Treatment Approaches and QoL Measurement

In our study, all patients received EBRT, and the majority also underwent brachytherapy, in line with current standards of care. Published evidence indicates that the combination of EBRT and brachytherapy contributes to improved local control and, importantly, better functional outcomes when compared to EBRT alone [19]. Our findings are consistent with this perspective, as patients maintained stable QoL during treatment and reported significant improvements afterwards. This suggests that, despite the intensified treatment burden of combined modalities, the functional benefits of brachytherapy integration may outweigh its acute toxicity, ultimately contributing to the favorable QoL trajectory observed in our cohort.

Additionally, cancer stage significantly affects QoL across various domains, underscoring the importance of early diagnosis and stage-appropriate treatment [32].

### 4.9. Emotional and Psychological Well-Being

In our study, Emotional and Psychological Well-Being declined slightly during treatment, but returned to baseline levels after completion of therapy, suggesting that the emotional impact of chemoradiotherapy was transient and reversible.

Emotional Well-Being often remains a challenge even after physical, social, and functional improvements post-treatment. Persistent psychological issues, such as anxiety, depression, and concerns about recurrence, affect patients, regardless of the treatment modality [29,33]. For instance, women undergoing CCRT frequently report negative impacts on body image, sexual enjoyment, and self-perception [34]. Emotional distress is further influenced by marital status, with single and widowed patients experiencing greater loneliness and less emotional support [21]. Depression, often linked to functional limitations and disrupted daily routines, also contributes to poor Emotional Well-Being [35,36].

Taken together, our results suggest that while Emotional and Psychological Well-Being remain recognized vulnerabilities in cervical cancer survivors, in our cohort, the immediate adverse effects of chemoradiotherapy were temporary, with patients regaining their baseline emotional status after treatment completion and at the first follow-up evaluation.

### 4.10. Long-Term Adaptations and Trends in QoL

Longitudinal studies reveal that treatment-related symptoms peak during therapy, while functional, social, and emotional domains often recover to pre-treatment levels by the end of treatment [21,37]. However, the persistent psychological and physiological stress caused by cancer and its treatment may continue to impair interrelated areas of health over the long term [38]. Some patients adapt to physical limitations, reporting perceived improvements in QoL despite ongoing physical challenges [39].

In line with these observations, our cohort demonstrated only a transient decline in QoL during chemoradiotherapy, with subsequent recovery and improvement across several domains, underscoring the need for continued longitudinal follow-up to determine whether these favorable short-term trajectories are sustained in the long term.

### 4.11. Study Limitations

This study has several limitations. While real-time data collection allows for accurate assessment, there is a possibility that participants who completed the QoL evaluations differ from those who opted out. Additionally, factors such as individual resilience, social support, and coexisting health conditions may not have been fully captured.

The Functional Assessment of Cancer Therapy-General (FACT-G) questionnaire, while validated, may not adequately address issues specific to cervical cancer treatment, particularly gynecologic health-related challenges. Side effects, such as changes in sexual health and gastrointestinal symptoms, may be underrepresented, potentially leading to an incomplete understanding of treatment’s impacts on daily life.

The demographic homogeneity of the sample also limits the generalizability of the findings. Most participants were from similar healthcare settings, which may not fully reflect the experiences of patients from diverse backgrounds or healthcare environments.

### 4.12. Future Directions

Future research should aim to include larger, more diverse participant groups to improve the generalizability of the findings. QoL tools tailored to address the unique challenges of cervical cancer patients would enhance our understanding of treatment impacts. Longitudinal studies assessing QoL at multiple intervals post-treatment could provide valuable insights into recovery trajectories and long-term outcomes. Exploring additional factors, such as mental health and social support systems, would further contribute to a comprehensive understanding of QoL in this population.

Chemoradiation can significantly impact QoL due to acute and late toxicities, but untreated patients may also face high symptom burdens from progressive disease. A comparison between a radio chemotherapy cohort versus an untreated cohort could help personalize care, balancing treatment intensity with patient priorities, especially in frail or inoperable cases, and should be taken into consideration as a valuable discussion in further research.

## 5. Conclusions

In this prospective cohort of cervical cancer patients treated with chemoradiotherapy, we observed no substantial deterioration in quality of life during treatment, followed by a significant improvement after therapy completion. With decreased variability and fewer outliers across all variables, the consistency of QoL outcomes increased after therapy, indicating that the majority of patients benefited similarly. All domains showed statistically significant improvements (*p* < 0.05), with physical and functional well-being showing the biggest influence (*p* < 0.001). These findings suggest that, in the short term, chemoradiotherapy does not negatively impact overall patient-reported outcomes and may be associated with post-treatment gains in physical and functional well-being. Our results contrast with reports of marked declines during therapy, underscoring the importance of supportive care measures that may mitigate acute treatment burden. Future studies with longer follow-up are warranted to determine whether these favorable short-term QoL trajectories are sustained over time.

## Figures and Tables

**Table 1:** Patient characteristics.

Characteristic	*n* = 111 ^1^
Age	58 (29–82, 12)
Marital status	
Divorced	13 (12%)
Married	70 (63%)
Not married	15 (14%)
Single	13 (12%)
Area	
Rural	37 (33%)
Urban	74 (67%)
Stage	
I	12 (11%)
II	38 (34%)
III	55 (50%)
IV	5 (4.5%)
Income	
High	7 (6.3%)
Low	26 (23%)
Middle	78 (70%)
Histology	
Adenocarcinoma	27 (24%)
Neuroendocrine	3 (2.7%)
Squamous cell	81 (73%)
Chemotherapy	
Carboplatin	8 (7.8%)
Cisplatin	94 (92%)
Unknown	9
Brachytherapy	110 (99%)

^1^ Mean (Min–Max, SD); *n* (%).

**Table 2:** QoL scores. FACTG—total score, EWB—Emotional Well-Being, FWB—Functional Well-Being, SWB—Social Well-Being, PWB—Physical Well-Being.

Characteristic	Before *n* = 111 ^1^	During *n* = 111 ^1^	After *n* = 111 ^1^	*p*-Value ^2^
FACTG	85 (73, 92)	84 (71, 93)	92 (84, 100)	<0.001
EWB	19.0 (15.0, 22.0)	20.0 (16.0, 22.0)	20.0 (18.0, 23.0)	0.004
FWB	20.0 (17.0, 24.0)	20.0 (17.0, 25.0)	23.0 (19.0, 27.0)	<0.001
SWB	24.0 (21.0, 27.0)	24.0 (21.0, 27.0)	25.7 (23.0, 28.0)	0.068
PWB	23.0 (17.0, 26.0)	21.0 (15.0, 24.0)	25.0 (21.5, 28.0)	<0.001

^1^ Median (Q1, Q3). ^2^ One-way analysis of means.

**Table 3:** Number and percentage of patients experiencing each toxicity, stratified by grade (Grades 1–4). Values are presented as *n* (%). Percentages represent the proportion within each grade (i.e., out of all patients who experienced Grade 1, 2, 3, 4.).

Grade ^1^	0 (%)	1 (%)	2 (%)	3 (%)	4 (%)
Toxicity					
Diarrhea	64 (9.7%)	38 (20%)	5 (17%)	1 (20%)	3 (75%)
Dysuria	77 (12%)	30 (16%)	3 (10%)	1 (20%)	0 (0%)
Emesis	75 (11%)	31 (17%)	4 (13%)	1 (20%)	0 (0%)
Hematological	94 (14%)	14 (7.5%)	2 (6.7%)	1 (20%)	0 (0%)
Mucositis	98 (15%)	10 (5.4%)	3 (10%)	0 (0%)	0 (0%)
Nausea	45 (6.8%)	51 (27%)	13 (43%)	1 (20%)	1 (25%)
Ototoxicity	103 (16%)	8 (4.3%)	0 (0%)	0 (0%)	0 (0%)
Renal	107 (16%)	4 (2.2%)	0 (0%)	0 (0%)	0 (0%)

^1^ *n* (%).

## Data Availability

The raw data supporting the conclusions of this article will be made available by the authors on request.

## Abbreviations

The following abbreviations are used in this manuscript:

AEs	Adverse Events
BT	Brachytherapy
CC	Cervical Cancer
CCRT	Concurrent Chemoradiotherapy
EBRT	External Beam Radiation Therapy
EWB	Emotional Well-Being
FACT-G	Functional Assessment of Cancer Therapy-General
FWB	Functional Well-Being
HPV	Human Papilloma Virus
HRQoL	Health-related quality of life
IMRT	Intensity-Modulated Radiation Therapy
PWB	Physical Well-Being
SWB	Social Well-Being
QoL	Quality of Life
